# Targeting stromal-induced pyruvate kinase M2 nuclear translocation impairs OXPHOS and prostate cancer metastatic spread

**DOI:** 10.18632/oncotarget.4448

**Published:** 2015-06-27

**Authors:** Elisa Giannoni, Maria Letizia Taddei, Andrea Morandi, Giuseppina Comito, Maura Calvani, Francesca Bianchini, Barbara Richichi, Giovanni Raugei, Nicholas Wong, Damu Tang, Paola Chiarugi

**Affiliations:** ^1^ Department of Experimental and Clinical Biomedical Sciences, University of Florence, 50134, Florence, Italy; ^2^ Department of Chemistry, University of Florence, 50019, Sesto Fiorentino, Italy; ^3^ Division of Nephrology, Department of Medicine, McMaster University, L8N4A6, Hamilton, Ontario, Canada

**Keywords:** pyruvate kinase M2, cancer associated fibroblasts, epithelial-mesenchymal transition, hypoxia inducible factor-1α, prostate cancer

## Abstract

Cancer associated fibroblasts (CAFs) are key determinants of cancer progression. In prostate carcinoma (PCa), CAFs induce epithelial-mesenchymal transition (EMT) and metabolic reprogramming of PCa cells towards oxidative phosphorylation (OXPHOS), promoting tumor growth and metastatic dissemination. We herein establish a novel role for pyruvate kinase M2 (PKM2), an established effector of Warburg-like glycolytic behavior, in OXPHOS metabolism induced by CAFs. Indeed, CAFs promote PKM2 post-translational modifications, such as cysteine oxidation and Src-dependent tyrosine phosphorylation, allowing nuclear migration of PKM2 and the formation of a trimeric complex with hypoxia inducible factor-1α (HIF-1α) and the transcriptional repressor Differentially Expressed in Chondrocytes-1 (DEC1). DEC1 recruitment is mandatory for downregulating miR205 expression, thereby fostering EMT execution and metabolic switch toward OXPHOS. Furthermore, the analysis of a cohort of PCa patients reveals a significant positive correlation between PKM2 nuclear localization and cancer aggressiveness, thereby validating our *in vitro* observations. Crucially, *in vitro* and *in vivo* pharmacological targeting of PKM2 nuclear translocation using DASA-58, as well as metformin, impairs metastatic dissemination of PCa cells in SCID mice. Our study indicates that impairing the metabolic tumor:stroma interplay by targeting the PKM2/OXPHOS axis, may be a valuable novel therapeutic approach in aggressive prostate carcinoma.

## INTRODUCTION

The acquisition of malignant traits of a cell during tumorigenesis is strongly influenced by the surrounding microenvironment [1, 2]. Among stromal cells, cancer-associated fibroblasts (CAFs) cross-talk with cancer cells to sustain tumor growth and enhance metastatic potential [3–5]. This bidirectional interplay has been reported in melanoma [6], breast [7] and prostate cancers (PCa) [8]. Particularly, we have shown that PCa cell-secreted IL-6 plays a pivotal role in promoting stromal reactivity. Once activated, CAFs release matrix metalloproteases 2 and 9 (MMP-2 and -9), eliciting a redox-dependent epithelial-to-mesenchymal transition (EMT) in PCa cells. This results in the enhancement of invasive abilities, acquisition of stem-like traits finally leading to metastatic dissemination [8, 9]. Recently, microRNA 205 (miR205) has been demonstrated to be a negative regulator of EMT and its expression inversely correlated with tumor malignancy [10, 11]. Indeed, PCa cells decrease miR205 expression upon CAFs contact, allowing de-repression of ZEB1/2 transcription factors and therefore promoting EMT [11].

Furthermore, CAFs and PCa cells establish a metabolic symbiotic cooperation: CAFs undergo Warburg metabolism and therefore increase glucose consumption whilst enhancing lactate production, which is released in the extracellular compartment via the mono-carboxylate transporter 4 (MCT4). Conversely, PCa cells reactivate oxidative phosphorylation (OXPHOS) and exploit CAF-derived lactate to fuel anabolic pathways and support cell growth even in glucose-free environment [12].

Pyruvate kinase (PK) controls the final and rate-limiting reaction of glycolysis. Although normal cells generally express the PKM1 isoform, tumor cells frequently switch to PKM2 expression [13, 14]. Particularly, PKM2 expression contributes to tumor cell metabolic alterations and is associated with Warburg metabolism [15, 16]. In contrast to PKM1, which is present in a constitutively tetrameric active form, PKM2 undergoes conformational conversion between a tetrameric/full active and a dimeric/less active state [17, 18]. A number of post-translational modifications have been reported to impair PKM2 enzymatic activity by promoting the tetramer to dimer conversion, including Tyr105 phosphorylation [19] and Cys358 oxidation [20]. Notably, the conversion to a less active state confers to PKM2 “non-metabolic” abilities. Indeed, PKM2 translocates into the nucleus and acts as a transcriptional co-activator of β-catenin and hypoxia-inducible factor 1α (HIF-1α), cooperating to control cell proliferation and glucose catabolism, respectively [21, 22].

We herein demonstrate that upon CAFs exposure, nuclear PKM2 controls EMT and metabolic reprogramming of PCa cells, thereby connecting motility and OXPHOS behavior. Once oxidized and phosphorylated, PKM2 translocates into the nucleus, interacts with HIF-1α and recruits the basic helix-loop-helix protein Differentially Expressed in Chondrocytes-1 (DEC1). This trimeric complex drives miR205 transcriptional suppression leading to EMT accomplishment and metabolic conversion to OXPHOS of PCa cells.

## RESULTS

### PKM2 in PC3 cells is oxidized and phosphorylated upon CAFs conditioning

**Figure 1:**
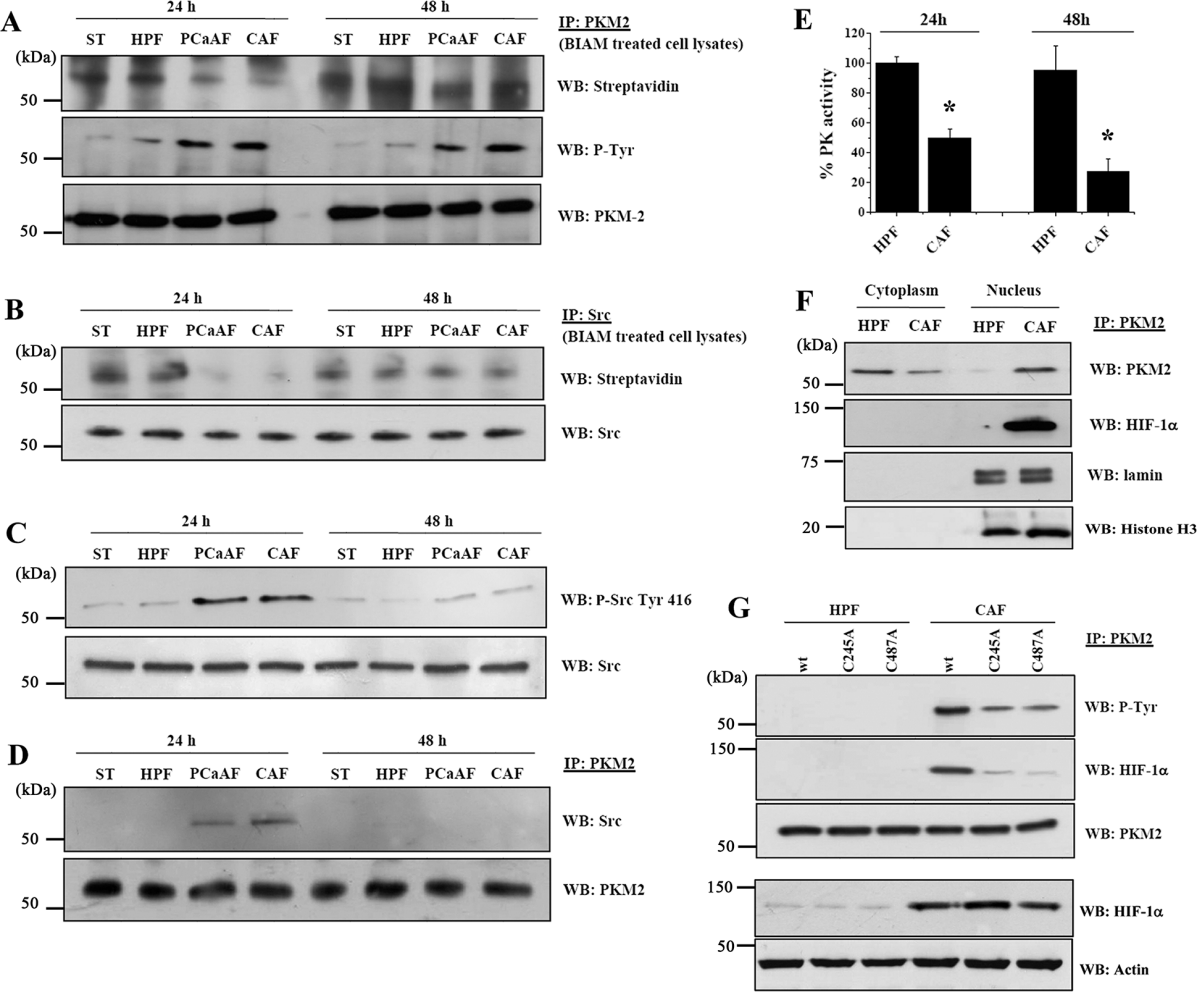
CAFs conditioning promotes PKM2 oxidation and tyrosine phosphorylation, leading to its nuclear localization and association with HIF-1α PC3 cells were cultured in serum-free medium (ST) or with CM from HPFs, PCaAFs (*in vitro* activated HPFs by PC3-CM) or patient-derived CAFs for 24 h or 48 h. **A.** PC3 cell were lysed in presence of BIAM and PKM2 was immunoprecipitated (IP). Western blot (WB) with HRP-streptavidin and with anti P-Tyr antibody were performed to evaluate the reduced form of PKM2 and its tyrosine phosphorylation, respectively. Anti-PKM2 antibody was used for normalization. **B.** Src kinase IP from BIAM-labeled lysates were subject to anti HRP-streptavidin (to reveal the reduced form of Src) and to anti-Src for normalization. **C.** Total cell lysates were subject to WB using the anti Src-Tyr 416 or anti Src antibodies. **D.** PKM2/Src association was revealed by WB analysis on PKM2 IP using an anti-Src antibody. PKM2 immunoblot was used for normalization. **E.** The analysis of PK activity was performed on PC3 cells after 24 h or 48 h of treatment with CM from HPFs or CAFs. *p < 0.005 *vs* HPF. **F.** Nuclear and cytosolic fractions derived from HPFs CM or CAFs CM 48 h treated PC3 cells were subject to PKM2 IP. Anti-HIF-1α and anti-PKM2 immunoblots were used to evaluate a direct association of the two proteins and for normalization, respectively. Lamin and histone H3 immunoblot were used as a control for nuclear extraction. **G.** The redox insensitive Src mutants (C245A and C487A) and Src wt plasmids were transfected into PC3 cells and after 24 h cells were incubated for additional 48 h with HPFs CM or CAFs CM. Total cell lysates were subject to PKM2 IP and WB analysis performed using the listed antibodies.

We have previously demonstrated that CAFs can induce cyclooxygenase 2-mediated oxidative stress in PC3 cells, leading to EMT execution [9]. Here, we show that 24 h administration of CAFs conditioned medium (CM) to PC3 cells leads to transient oxidation of PKM2, which reverts to the reduced state after 48 h of CAFs conditioning (Figure 1A). A similar result was obtained by treating PC3 cells with human prostate fibroblasts (HPFs) activated *in vitro* by exposure to CM derived from PCa cells (PCaAF) (Figure 1A). In addition to reversible oxidation, PKM2 undergoes a tyrosine phosphorylation in response to CAFs exposure that is observed both at 24 and 48 h (Figure 1A). The tyrosine kinase Src is a known redox sensor already reported to play a role in PCa metastatic behavior [23, 24]. Therefore, we analyzed Src redox state upon CAFs contact: CAFs-induced oxidative burst leads to Src oxidation (Figure 1B) that is paralleled by an increase in Src Y416 phosphorylation (Figure 1C), an indication of Src kinase activation. Moreover, Src associates with PKM2 following CAFs conditioning, suggesting a direct Src involvement in PKM2 phosphorylation (Figure 1D). In keeping with previous reports, oxidation and phosphorylation of PKM2 induce the destabilization of the tetrameric complex and the conversion towards a dimeric less metabolic active state (Figure 1E).

### CAFs induce PKM2 nuclear localization and association with HIF-1α

It is established that PKM2 in its dimeric state can translocate into the nucleus and exert a non-metabolic function [18]. Accordingly, CAF-induced PKM2 oxidation and phosphorylation allows its translocation into the nucleus, where PKM2 associates with the transcriptional factor HIF-1α (Figure 1F). To confirm the role of Src kinase in ensuring PKM2 phosphorylation/inactivation we transfected PC3 cells with the redox insensitive Src mutants (Src C245A and C487A) [23]. Ectopic expression of Src C245A and Src C487A strongly impairs PKM2 tyrosine phosphorylation and association with HIF-1α (Figure 1G), although the nuclear localization of the enzyme is unaffected (Supplementary Figure S1). Conversely, HPF-CM did not have any major effects on PKM2 function and localization (Figure 1A–1G), suggesting an exclusive role for CAFs.

### DASA-58-mediated PKM2 reactivation prevents its nuclear localization and association with HIF-1α, thereby blocking EMT

**Figure 2:**
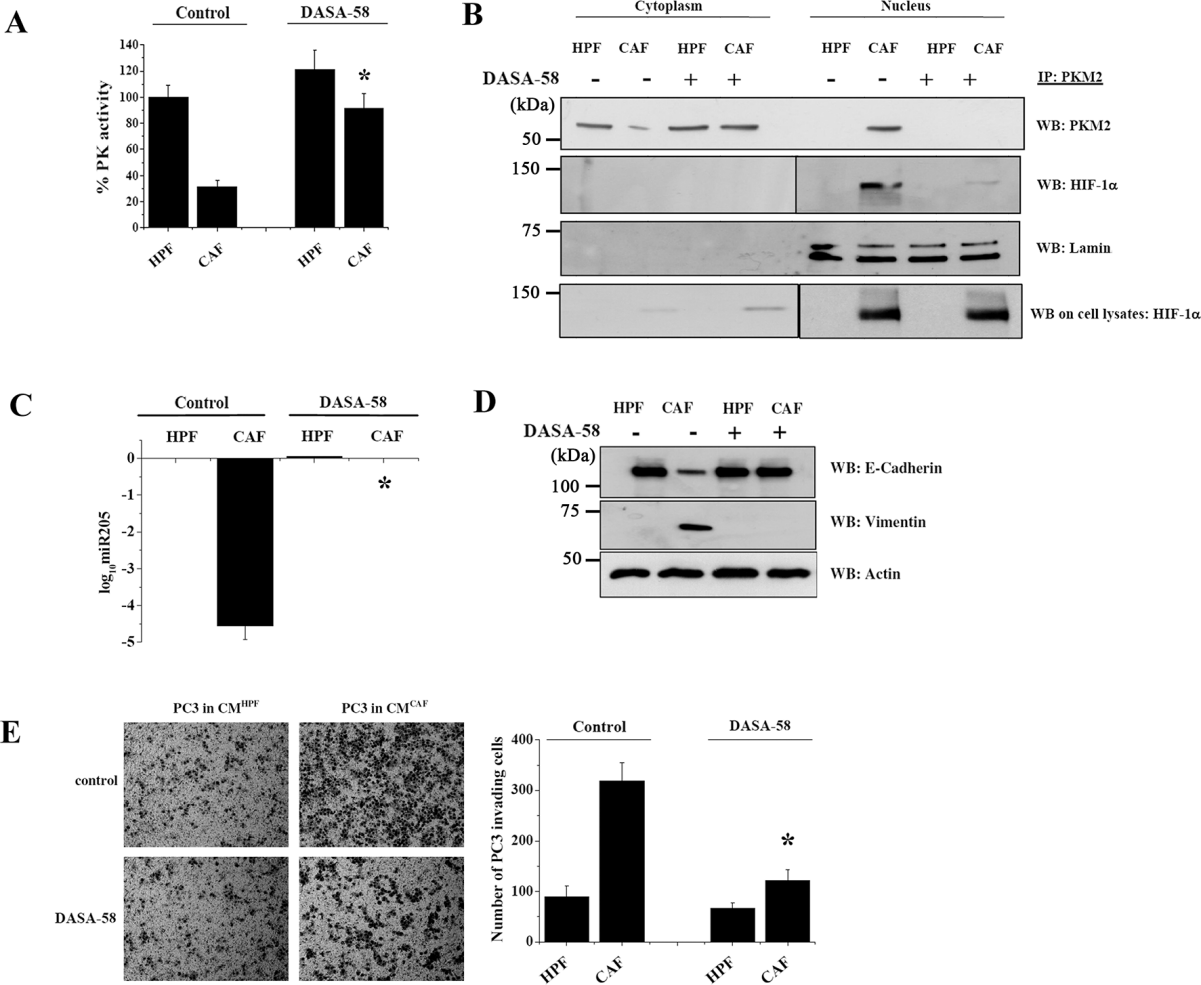
DASA-58 administration re-activates PKM2, impairs PKM2/HIF-1α nuclear association and abrogates EMT in PC3 cells PC3 cells were cultured with CM from HPFs or CAFs for 48 h with or without 40 μM DASA-58. **A.** Measure of PK enzymatic activity. *p < 0.005 *vs* control CAF **B.** PKM2 IP from both nuclear and cytosolic fractions were subjected to WB for anti-HIF-1α to evaluate PKM2/HIF-1α association. Anti PKM2 was used for normalization and anti-lamin as a control for nuclear extraction. Total amounts of HIF-1α on cell lysates were quantified by WB. **C.** miR-205 expression levels were quantified by qRT-PCR. Data are reported as log_10_-transformed relative quantity with respect to HPF-CM-PC3 treated cells. SNORD61 was used as comparator. *p < 0.001 *vs* control CAF **D.** EMT markers were monitored by WB with anti E-cadherin and anti vimentin antibodies. Actin was used for normalization. **E.** Invasion assay using Boyden chamber. Left, representative pictures are shown; right, bar graphs show the mean+SEM of the number of invading cells. For quantification six randomly chosen fields were counted. *p < 0.001 *vs* control CAF.

To unravel the role of the CAF-induced PKM2 inactivation and nuclear localization in PC3 cells, we analyzed the effect of synthetic PKM2 reactivation induced by DASA-58, which enhances PKM2 activity by inducing the tetramer formation [17]. Administration of DASA-58 during CAFs conditioning of PC3 cells is able to restore PK activity (Figure 2A), thereby abrogating the nuclear translocation of PKM2, as well as its association with HIF-1α (Figure 2B). Moreover, the impairment of PKM2/HIF-1α association significantly affects the expression of miR205, a critical miRNA driving CAF-mediated EMT in PCa cells [11] (Figure 2C). Indeed, miR205 downregulation induced by CAFs exposure is completely counteracted by DASA-58 treatment (Figure 2C). Interfering with PKM2 enzymatic activity by DASA-58 treatment impairs stromal-induced EMT program as indicated by both the analysis of established EMT markers (Figure 2D) and PC3 cells invasiveness (Figure 2E).

### PKM2 reactivation restores glucose dependency of PC3 cells, still sustaining OXPHOS

**Figure 3:**
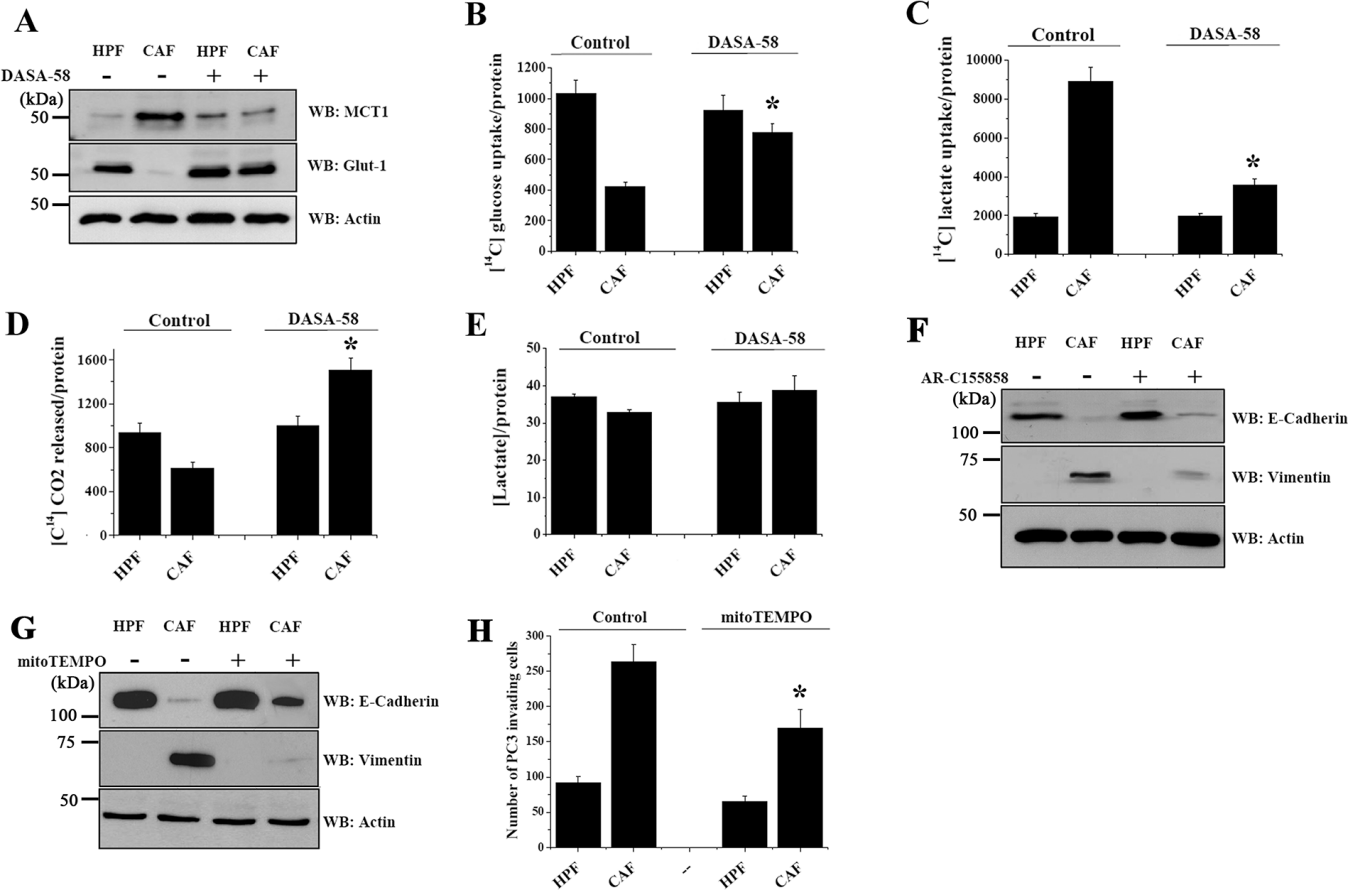
PKM2 reactivation induced by DASA-58 restores glucose respiration, impairing CAFs driven lactate consumption PC3 cells were cultured with CM from HPFs or CAFs for 48 h with or without 40 μM DASA-58 and subjected to different assays. **A.** The levels of the transporters MCT1 and Glut-1 were evaluated by immunoblotting. Actin immunoblot used for normalization is the same of Figure 2D, because it derives from the same experiment. **B–C.** Evaluation of [^14^C]-glucose (B) and [^14^C]-lactate uptake (C) were performed and normalized on protein content. **D.** Respiration of [^14^C]-glucose was evaluated as [^14^C]-CO_2_ release and normalized on protein content. **E.** PCa cells were cultured with CM from HPFs or CAFs for 48 h. Then media were changed with a serum-free one for additional 16 h with or without 40 μM DASA-58. The amount of lactate released by PC3 cells was quantified and normalized on cell protein content. **F–G.** PC3 cells were cultured with CM from HPFs or CAFs for 48 h with or without 1 μM AR-C155858 (F) and 50 mM MitoTEMPO (G). EMT markers were analyzed by WB with anti E-cadherin and anti vimentin antibodies. Actin was used for normalization. **H.** PC3 cells were treated as in G and an invasion assay using Boyden chamber was then performed. Bar graphs show the mean+SEM of the number of invading cells. For quantification six randomly chosen fields were counted. *p < 0.005 *vs* control CAF.

We have previously shown a reciprocal metabolic reprogramming between CAFs and PCa cells. CAFs converted to Warburg metabolism, release lactate which is rapidly uptake by PCa cells to fuel metabolic pathways and OXPHOS, sustaining their energetic needs and growth [12]. Here, we investigated the effect of PKM2 reactivation on this metabolic circuitry. DASA-58 administration severely impairs CAF-induced upregulation of MCT1 and prevents CAF-induced downregulation of the glucose transporter Glut-1 (Figure 3A). Accordingly, DASA-58 reactivation of PKM2 restores PC3 cells glucose consumption (Figure 3B) while impairing their ability to import CAF-derived lactate (Figure 3C). Despite DASA-58 administration is able to convert PC3 cells from a lactate-dependent to a glucose-dependent metabolism, the OXPHOS ability of PC3 cells is unaffected. Indeed, DASA-58 treatment during CAFs conditioning promotes a significant increase of glucose respiration (Figure 3D). Conversely, DASA-58 does not significantly impact on lactate extrusion of PC3 cells (Figure 3E). Therefore, DASA-58-induced PKM2 metabolic reactivation is sufficient to abolish the energetic symbiosis between PCa cells and CAFs, to restore glucose dependency in cancer cells, while maintaining their OXPHOS phenotype.

The ectopic expression of the redox insensitive Src C245A and Src C487A, which abrogates PKM2 association with HIF-1α without interfering with its nuclear translocation, induces an effect comparable to DASA-58 administration. Indeed, expression of Src mutants reduces lactate uptake (Supplementary Figure S2A) and restores glucose consumption and respiration (Supplementary Figure S2B, S2C) without affecting lactate production and extrusion (Supplementary Figure S2D).

In order to clarify if the CAF-induced increase in lactate import, *via* MCT1 upregulation, could be responsible for EMT initiation in PC3 cells, we investigated the effect of the MCT1 inhibitor, AR-C155858. The inhibition of the lactate importer significantly impairs EMT, suggesting a crucial role of lactate in the acquisition of PC3 pro-invasive features (Figure 3F). In keeping with the recent evidence linking mitochondrial ROS production to the enhancement of MCT1 expression and tumor cell migration [25, 26], we also show that interfering with mitochondrial ROS generation, using the mitochondria-targeted antioxidant MitoTEMPO, impairs EMT accomplishment and PC3 invasiveness upon CAF-conditioning (Figure 3G–3H).

### CAFs induce the recruitment of the transcriptional repressor DEC1 to PKM2/HIF-1α complex

**Figure 4:**
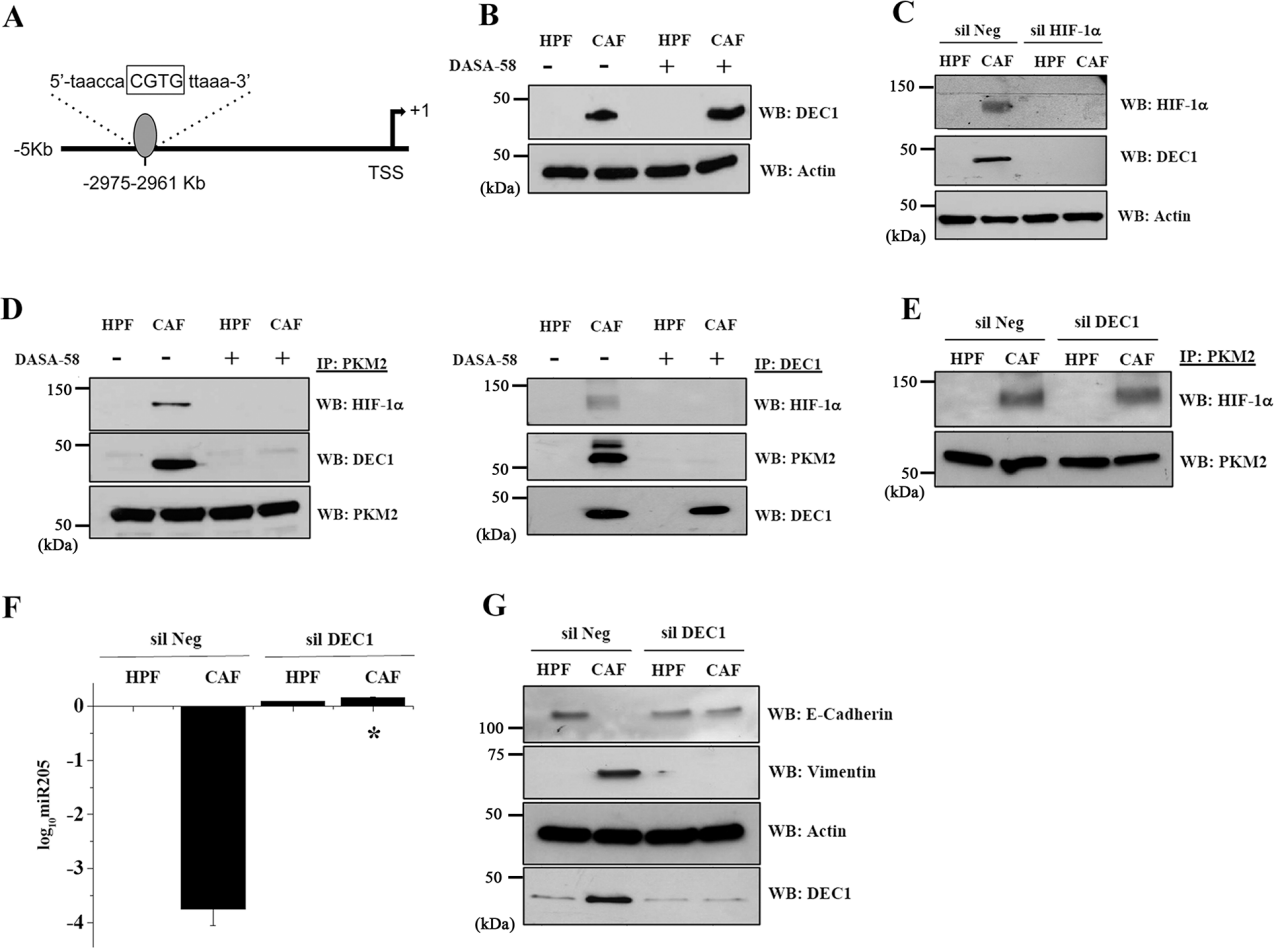
DEC1 is recruited by the PKM2/HIF-1α complex upon CAFs exposure, granting for miR205 transcriptional repression and EMT execution **A.** Schematic representation of putative DEC1 binding site within the miR205 promoter region identified using Genomatix Suite. The putative sequence is indicated and the core sequence (four most conserved positions within the matrix) is highlighted in capital letter. **B.** PC3 cells were cultured with CM from HPFs or CAFs for 48 h with or without 40 μM DASA-58. DEC1 expression level was evaluated by WB with anti DEC1 antibody (DEC1 (S-8), sc-101023, Santa Cruz Biotechnology). An actin WB was used for normalization. **C.** HIF-1α was silenced in PC3 cells and after 24 h cells were incubated for additional 48 h with HPFs CM or CAFs CM (two different siRNA from Santa Cruz Biotechnology and Origene were used, with similar results). DEC1 and HIF-1α levels were evaluated by WB. Actin immunoblot was used for normalization. **D.** PC3 cells were treated as in B. (Left panel) PKM2 was immunoprecipitated and anti-HIF-1α and anti-DEC1 WB were performed to evaluate a direct association with PKM2. PKM2 immunoblot was used for normalization. (Right panel) DEC1 was immunoprecipitated and anti-HIF-1α and anti-PKM2 immunoblots were performed to assess the binding with DEC1. DEC1 immunoblot was used for normalization. **E.** DEC1 was silenced in PC3 cells and after 24 h cells were incubated for additional 48 h with HPFs CM or CAFs CM (two different siRNA from Santa Cruz Biotechnology and Origene were used, with similar results). PKM2/HIF-1α association was assessed by an anti-HIF-1α WB on PKM2 IP. PKM2 immunoblot was used for normalization. **F.** PC3 cells were treated as in E. qRT-PCR analysis of miR-205 expression is reported as log_10_-transformed relative expression with respect to HPF-CM-PC3 treated cells. *p < 0.001 *vs* silNeg CAF **G.** The levels of E-cadherin and vimentin were assessed by immunoblotting. The anti-actin WB was used for normalization, while the anti-DEC1 was performed as a control of silencing.

To clarify the molecular mechanism that control miR205 expression, we evaluated whether other transcriptional regulators could be recruited at the PKM2/HIF-1α complex. We focused our interest on DEC1 (BHLHE40/STRA13/SHARP-2), a transcriptional repressor under the control of HIF-1α, already demonstrated to be involved in cancer aggressiveness and motility [27, 28]. Interestingly, *in silico* analysis of the miR205 promoter region by Genomatix (www.genomatixsuite.de), revealed one putative DEC1 binding site on the 3 kb proximal promoter of the MIR205HG sense strand at −2975 bp −2961 bp (Figure 4A). In addition, we confirmed that CAFs conditioning induces DEC1 expression in PC3 cells and that this regulation is not affected by DASA-58 administration (Figure 4B). Silencing of HIF-1α before CAFs CM administration completely abrogates DEC1 upregulation, confirming that HIF-1α is responsible for DEC1 induction upon CAFs CM exposure (Figure 4C). To assess whether DEC1 could be involved in prostate tumor:stroma interplay, we investigated the ability of DEC1 to cooperate with PKM2 to drive the motogen pathway in PC3 cells. We found that PKM2 associates with both HIF-1α and DEC1 and that DASA-58 administration (i.e. impairment of PKM2/HIF-1α complex) interferes with DEC1 recruitment (Figure 4D left and right panels). Notably, DEC1 silencing does not affect the PKM2/HIF-1α complex formation (Figure 4E). In keeping with its role of transcriptional repressor, *de novo* expression of DEC1 and the formation of the trimeric complex DEC1/PKM2/HIF-1α is mandatory for allowing miR205 downregulation after CAFs conditioning (Figure 4F) and for EMT induction (Figure 4G). The transcriptional repressive function of DEC1 is probably responsible also for the CAF-mediated reduction in glucose consumption via downregulation of the transporter Glut-1. Indeed, in silico analysis of Glut-1 promoter region unravels 4 putative DEC1 binding sites (Supplementary Figure S3A). In agreement, DEC1 silencing impairs Glut-1 downregulation in CAF-conditioned PC3 cells, thereby reactivating glucose import (Supplementary Figure S3B–S3C).

To further support the transcriptional repressive role of DEC1 in our model, we investigated the effect of the redox insensitive Src mutants (Src C245A and Src C487A) on DEC1/PKM2 association and the consequential modulation of miR205 expression. Since the redox insensitive Src mutants impair PKM2 phosphorylation and association with HIF-1α, the recruitment of DEC1 is also impaired (Supplementary Figure S4A). The absence of the PKM2/HIF-1α/DEC1 complex does not allow PC3 cancer cells to downregulate miR205 expression once treated with CAFs CM (Supplementary Figure S4B).

DEC1 association with the PKM2/HIF-1α complex was also confirmed in DU145 cells, another metastatic prostate carcinoma cell line (Supplementary Figure S5A). As already observed in PC3 cells, DASA-58 administration abrogates the CAF-induced miR205 downregulation in DU145 cells (Supplementary Figure S5B), thereby interfering with the EMT execution and the pro-invasive input elicited by CAFs (Supplementary Figure S5C–S5E).

### Metformin abolishes the PKM2-mediated EMT commitment induced by CAFs

**Figure 5:**
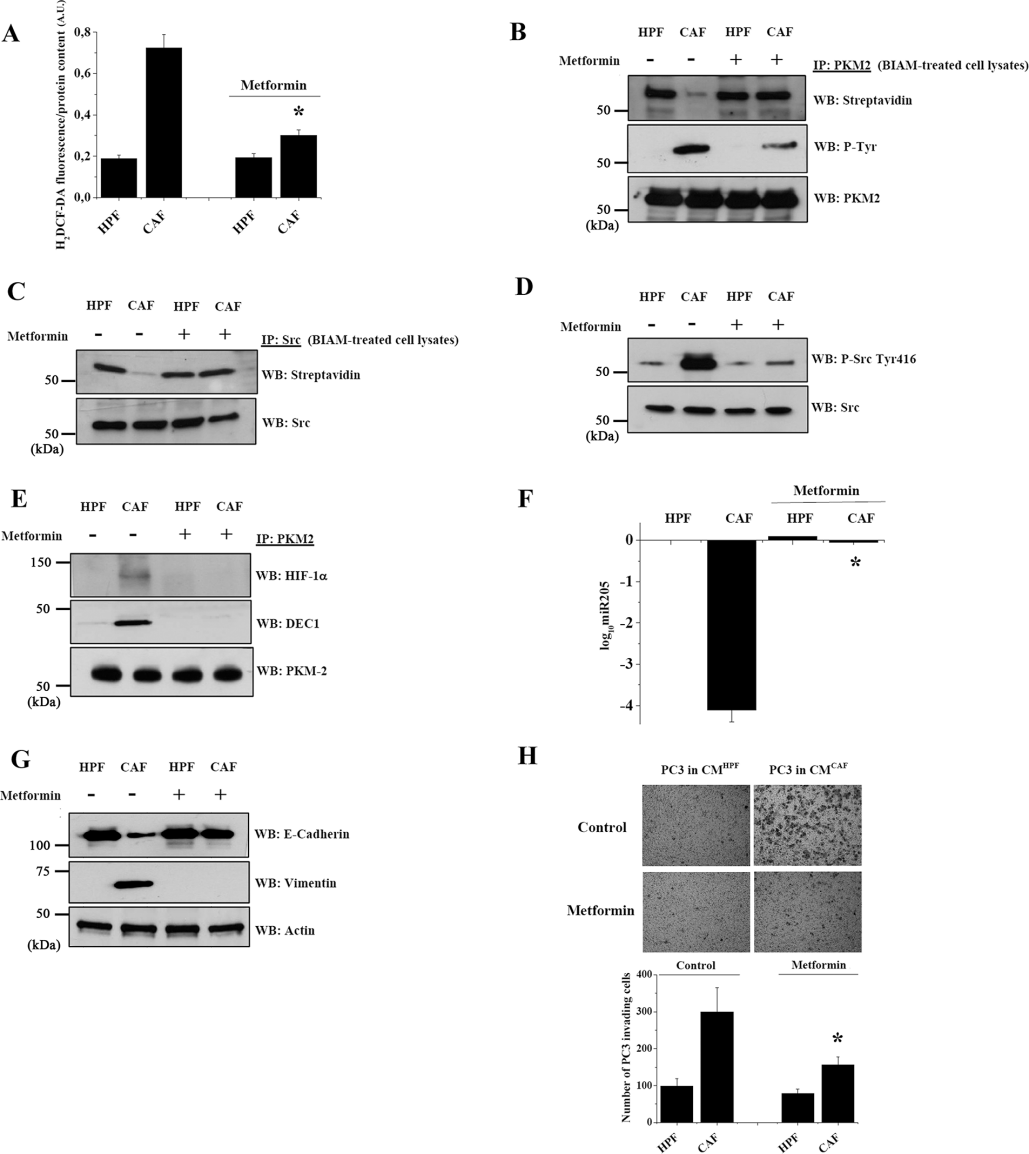
Metformin impairs PKM2 nuclear function and abrogates CAFs driven EMT in PC3 cells PC3 cells were cultured with CM from HPFs or CAFs for 48 h with or without 5 mM metformin. **A.** H_2_O_2_ production was evaluated by H_2_DCF-DA spectrophotometric analysis, normalized on protein content. *p < 0.005 *vs* control CAF **B.** PKM2 IP from BIAM-labeled cell lysates were subjected to HRP-streptavidin, anti-P-Tyr and anti-PKM2 WB to evaluate the reduced form, the tyrosine phosphorylation and the total amount of PKM2, respectively. **C.** Src kinase was immunoprecipitated from BIAM-labeled lysates and the reduced form of Src was revealed by anti HRP-streptavidin WB. Src immunoblot was performed for normalization. **D.** Src activation was assessed by anti Src-Tyr 416 immunoblot and normalized on Src total amount. **E.** PKM2 was immunoprecipitated from cell lysates and anti-HIF-1α and anti-DEC1 WBs were performed to evaluate a direct association with PKM2. PKM2 immunoblot was used for normalization. **F.** qRT-PCR analysis of miR-205 expression is shown as log_10_-transformed relative expression compared to HPF-CM-PC3 treated cells. *p < 0.001 *vs* control CAF **G.** Expression of E-cadherin and vimentin was evaluated by WB. Actin immunoblot was used as loading control. **H.** Invasion assay using matrigel-coated Boyden chamber was performed using PC3 cells treated as indicated. Representative pictures are shown and quantification of six randomly chosen fields (*n* = 3) is reported in bar graphs as mean+SEM. *p < 0.001 *vs* control CAF.

Recent observations demonstrated a clear role of the anti-diabetic drug metformin in the reduction of primary tumor growth and metastatic colonization in several solid tumors [29]. In keeping with the established effect of metformin in inhibiting complex I of the mitochondrial electron transport chain [30], we observed that metformin reduces ROS levels in PC3 cells after CAFs conditioning (Figure 5A) and negatively affects both the oxidation and tyrosine phosphorylation of PKM2 (Figure 5B). The reduction of PKM2 phosphorylation is due to the metformin inhibitory effect on Src oxidation and kinase activation (Figure 5C, 5D). Metformin counteracts PKM2 inactivation and therefore i) impairs the formation of the HIF-1α/DEC1/PKM2 trimeric complex (Figure 5E), ii) downregulates CAF-induced *miR205* expression (Figure 5F), iii) inhibits CAF-induced EMT promotion (Figure 5G) and subsequent cells invasive ability (Figure 5H). Comparable results were obtained in DU145 cells, confirming the ability of metformin to inhibit PKM2 nuclear translocation and CAF-induced inhibition of miR205 expression, thereby impairing EMT execution (Supplementary Figure S6A–S6D).

### Metformin abolishes the metabolic cooperation between CAFs and PC3 cells, converting cancer cells to a Warburg phenotype

**Figure 6:**
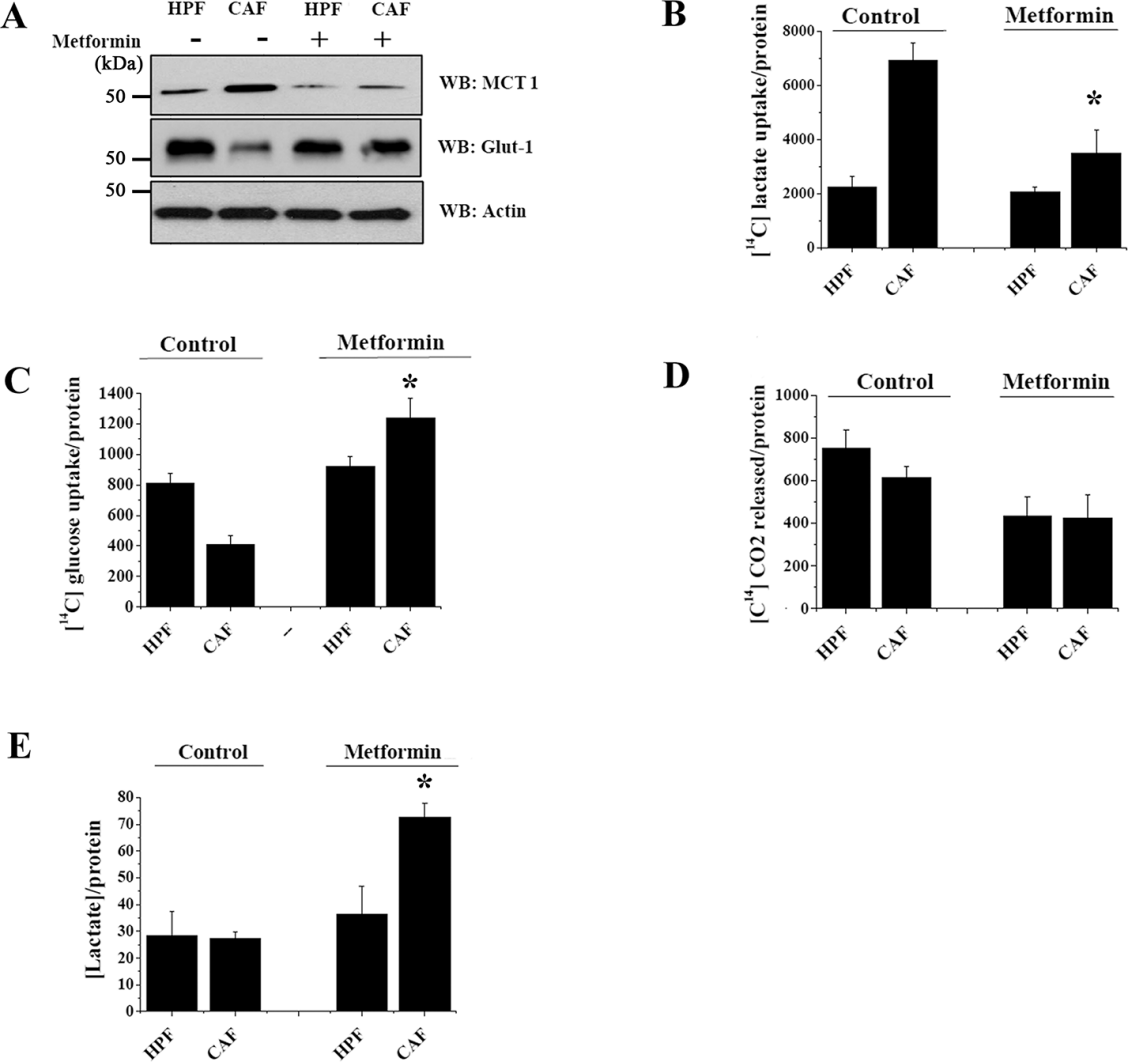
Metformin increases glucose consumption in PC3 cells exposed to CAFs CM PC3 cells were cultured with CM from HPFs or CAFs for 48 h with or without 5 mM metformin. **A.** The levels of MCT1 and Glut-1 were evaluated by WB and actin WB was used as loading control. **B–C.** Evaluations of [^14^C]-lactate (B) and [^14^C]-glucose uptake (C) were performed and normalized on total proteins. **D.** Respiration of [^14^C]-glucose was evaluated as [^14^C]-CO_2_ release and normalized on protein content. **E.** PCa cells were cultured with CM from HPFs or CAFs for 48 h. Then media were changed with a serum-free one for additional 16 h with or without 5 mM metformin. The amount of lactate released by PC3 cells was quantified and normalized on protein content. *p < 0.005 *vs* control CAF.

Similar to what is observed for DASA-58, metformin administration regulates PKM2 nuclear function of PC3 cells impacting on their metabolic pathways. Indeed, metformin treatment of CAFs-conditioned PC3 cells reduces MCT1 and increases Glut-1 expression levels, thus promoting PC3 cells to shift their metabolic dependency from lactate to glucose (Figure 6A–6C). Since metformin prevents OXPHOS by inhibiting mitochondrial respiratory chain complex I (Figure 6D), it forces PC3 cells to recover a “canonical” Warburg metabolism, increasing lactate extrusion and disrupting the metabolic symbiosis of PC3 cells with stromal CAFs (Figure 6E).

### Impairment of PKM2 nuclear association with HIF-1α and DEC1 abrogates metastatic colonization of PC3 cells

**Figure 7:**
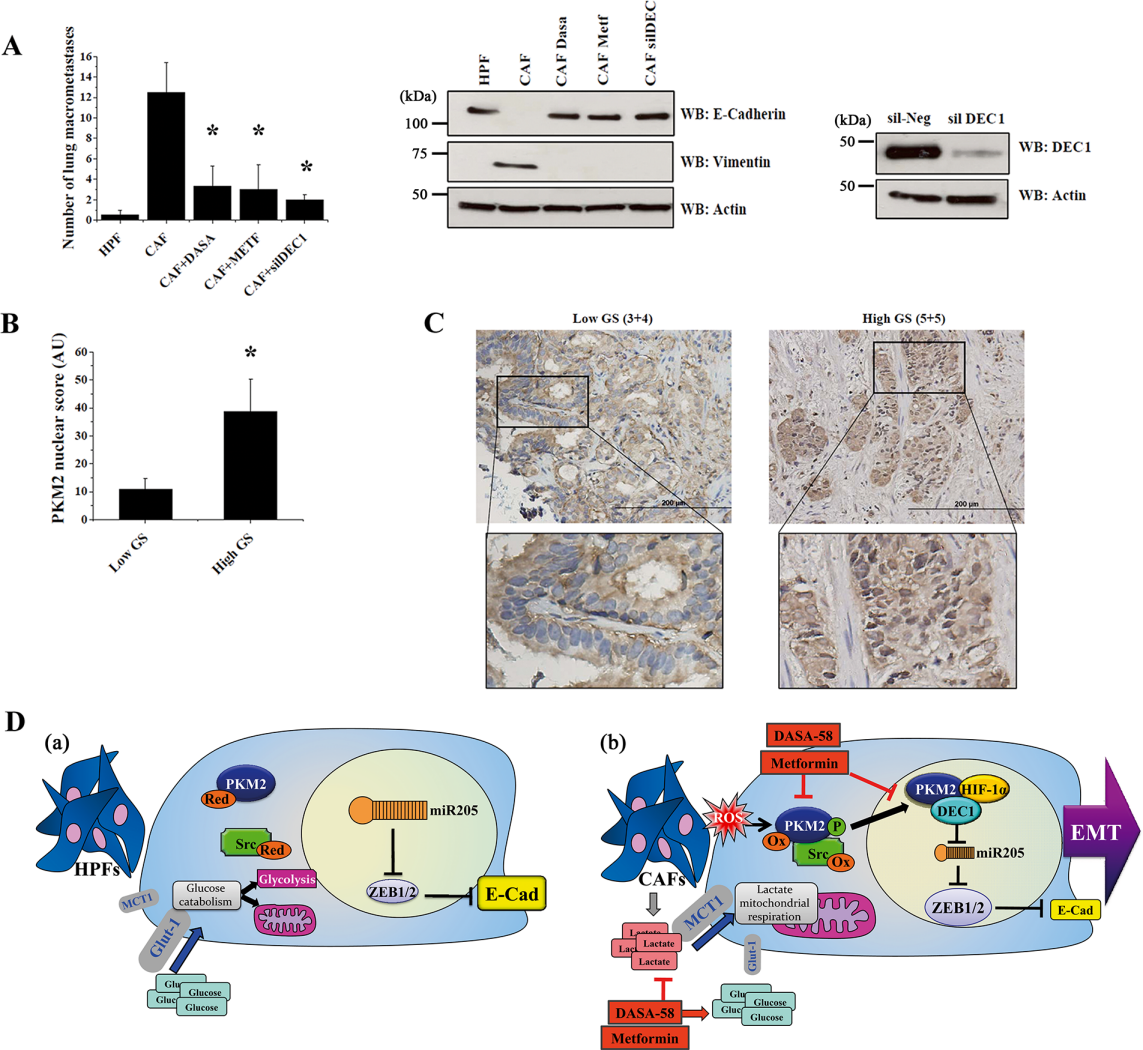
Nuclear translocation of PKM2 correlates with prostate carcinoma aggressiveness and with metastatic potential of PC3 cells **A.** PC3 lung colonization in SCID bg/bg mice. PC3 cells were conditioned for 48 h with CM from HPFs or with CM from CAFs, with or without 40 μM DASA-58 or 5 mM metformin. Alternatively, PC3 cells were conditioned for 48 h with CAFs CM after 24 h of DEC1 silencing. Treated cells were then injected into the lateral tail vein of mice (*n* = 6 per group). Lung macrometastases were counted (*p < 0.001 *vs* control CAF). Immunoblot for E-cadherin and Vimentin and for DEC1 expression were performed as a control of EMT and DEC1 silencing, respectively. **B.** 29 primary prostate cancer tissues with different Gleason scores (Low GS corresponding to Gleason scores 6–7 and High GS corresponding to Gleason scores 8–10) were IHC stained with anti-PKM2 antibody to evaluate PKM2 nuclear localization. The IHC staining was quantified based on the intensity and percentage of cells stained (see Materials and Methods for details). Bars represent mean +SEM (standard error of the mean) for Low and High GS *p < 0.05 *vs* Low GS **C.** Representative images for GS 3+4 (Low GS) and GS 5+5 (High GS) are shown. Scale bars = 200 *μ*m. The marked areas are higher magnification of the highlighted areas (3X). **D.** Schematic representation of PKM2 central role in PCa cells upon HPFs or CAFs exposure. **a.** During HPFs conditioning, PC3 cells upload glucose by the microenvironment, mainly used to fuel glycolysis, with a little involvement of mitochondrial respiration. HPF-treated PC3 cells also maintain high levels of miR205, thus allowing the stabilization E-cadherin-mediated epithelial features. **b.** The pro-oxidant environment induced by CAFs drives the oxidation and the Src-mediated tyrosine phosphorylation of PKM2, promoting its nuclear association with HIF-1α/DEC1, downregulation of miR205 and EMT execution. In addition, PKM2 nuclear translocation favors the establishment of a metabolic circuitry between CAFs and PCa cells. The impairment of PKM2 nuclear function with DASA-58 or metformin administration abolishes EMT, motility and the metabolic dependence of PCa cells on CAF-derived lactate, restoring glucose-dependency of cancer cells.

To confirm that CAF-induced PKM2 nuclear translocation is an essential requirement for PCa cells metastatic ability, we performed an experimental metastasis assay in SCID mice. PC3 cells where either silenced for DEC1 prior to CAFs conditioning or cultured in presence of CAFs-CM with or without DASA-58 or metformin. SCID mice were i.v. injected with *ex vivo* treated cells and analysis of lung colonization was performed after 40 days from tail vein injection. Before injection, we checked the efficacy of drugs administration or DEC1 silencing in abrogating EMT (Figure 7A, right panel). CAFs conditioning of PC3 cells significantly increased (12-fold) lung metastatic nodules formation compared to HPF-treated PC3 (Figure 7A, left panel). Crucially, silencing of DEC1, as well as the treatment with DASA-58 or metformin, dramatically reduced (∼6-fold) CAFs-induced lung metastases formation (Figure 7A, left panel).

### Nuclear localization of PKM2 correlates with PCa malignancy *in vivo*

To further corroborate our *in vitro* and *in vivo* observations, we evaluated the intensity of PKM2 nuclear staining in prostate specimens derived from patients affected by PCa with different Gleason score (GS). Our analysis reveals a significant increase (*P* < 0.05) of PKM2 nuclear localization in prostate carcinomas with higher (8–10) versus lower Gleason scores (6–7) (Figure 7B, 7C). Together, our data demonstrate that CAFs promoted conversion of PCa cells towards a highly aggressive and metastatic phenotype is controlled by PKM2 transition towards a less metabolically active state and the subsequent acquisition of a “non-metabolic” nuclear activity.

## DISCUSSION

In the current manuscript, we highlight a novel function of PKM2 besides its established role in controlling the Warburg metabolic deregulation of cancer cells. Importantly, we demonstrate that PKM2 is a master regulator of cancer cell motility induced by CAFs contact. In fact, CAFs promote PCa progression, enabling cancer cells to successfully colonize and generate metastatic disease to distant sites. Crucially, in response to CAFs contact, PKM2 undergoes metabolic inactivation through both oxidation and Src-mediated phosphorylation inducing PKM2 nuclear translocation. Once in the nucleus, PKM2 can associate with HIF-1α and the transcriptional repressor DEC1, thus culminating in miR205 regulation, EMT execution and enhancement of invasiveness (Figure 7D a,b). Finally, the *in vitro* data are fully supported by *in vivo* observations in both SCID mice and PCa patients-derived specimens (Figure 7C). Therefore, PKM2 plays an essential role in mediating both functions exerted by CAFs for cancer malignancy, i.e. enhance cell motility and control metabolic reprogramming of PCa cells.

PKM2 expression correlates with cancer aggressiveness [13, 31–33] and represents the glycolysis bottleneck in cells undergoing Warburg metabolism (i.e. increased glucose uptake) [15, 19, 20]. Indeed, PKM2 enzymatic activity can be functionally controlled by several post-translational modifications [34]: PKM2 activity inhibition induces cancer cells to accumulate glycolytic intermediates that fuel aminoacid biosynthesis, pentose phosphate pathway and nucleotides anabolism, resulting in cancer growth promotion [15]. Although cancer cells may exploit different mechanisms to interfere with the glycolytic flux and fuel anabolism [35], PKM2 is widely considered the leading regulator of the Warburg metabolism in cancer cells. Here, we involve PKM2 as a modulator of the lactate-addicted respiratory metabolism induced by CAFs-conditioning in highly stroma-infiltrated cancers, thereby expanding the functions controlled by this enzyme from glycolysis regulation to OXPHOS. In prostate or breast carcinomas, CAFs display a canonical Warburg behavior and secrete lactate [12, 36]. Conversely, cancer cells upload CAFs-produced lactate to sustain mitochondrial OXPHOS, anabolic pathways and cell growth [12]. Treatment with DASA-58, which interferes with CAFs-induced PKM2 functional (i.e. enzymatic) inhibition, induces loss of lactate addiction and restores glucose dependency in cancer cells, although maintaining a respiratory phenotype sustained by glucose respiration. It is therefore intriguing to suggest that DASA-58 may be used for tumor:stroma metabolic coupling disruption. Particularly, its activity in highly stromal-infiltrated cancers may be further enhanced by OXPHOS inhibitors co-administration.

CAFs-induced EMT is a redox dependent phenomenon, driven by Rac1b/cycloxygenase-2 activation, which promotes the stabilization of the redox sensitive transcription factor HIF-1α, in turn sustaining a second wave of transcriptional activity due to ZEB1/2 and Snail-1 [9]. We now include both Src and PKM2, as direct targets of the CAF-induced pro-oxidant environment. Both these enzymes have already been reported as redox sensors [37]. PKM2 has been found oxidized in response to hypoxia and insulin stimulation, while Src is oxidized during hypoxia [38], upon cell adhesion to extracellular matrix (ECM) and in EGFR-mediated *anoikis* resistance [23, 24]. To our knowledge, this is the first report describing the concomitant redox regulation of both kinases, during CAFs-driven pro-inflammatory pathway that sustains metastases in PCa. Both regulations are functionally linked and collectively lead to PKM2 conversion to a less active state. In addition, in our model the CAF-induced tyrosine phosphorylation of PKM2 results from a direct association with Src. This is a novel finding, since Src has not been previously correlated to PKM2 phosphorylation.

Interestingly, our data also highlight a crucial role of PKM2 as a common regulator of both the motile and the metabolic features of cancer cells. The role of PKM2 in tumor cells is controverted. Besides its role in promoting cancer growth, Israelsen and colleagues reported that PKM2 genetic knock out increased breast tumorigenesis in a mouse model [39]. Herein, we show that PKM2 role in PCa progression is not merely associated to its expression, which is unaffected by CAFs conditioning, but also with the post-translational modifications induced by CAFs. These modifications are mandatory for PKM2 translocation into the nucleus, association with HIF-1α and DEC1 and induction of miR205-dependent EMT. In keeping with our data, Hamabe et al. recently reported a direct involvement of nuclear PKM2 in fostering TGFβ-mediated EMT in colon cancer cells. The authors propose a role for PKM2 as a transcription cofactor of TGF-β-induced factor homeobox 2 in driving histone H3 deacetylation and inhibition of *CDH1* expression [40].

Notably, our study indicates a positive correlation between nuclear PKM2 localization and prostate cancer aggressiveness in patient-derived tumor tissues. Nuclear migration of PKM2 has already been reported in several studies [21, 22], involving different mechanisms such as serine or tyrosine PKM2 phosphorylation and association of PKM2 with prolyl hydroxylases and JMJ demethylases [32, 41]. In this still evolving *scenario*, one possibility is that the controversial role of PKM2, that we proved to be importantly influenced by contact between cancer cells and CAFs, can be explained by different grades of stromal infiltration in different histotypes of cancers.

Our data indicate that both DASA-58 and metformin similarly affect EMT of prostate cancers and tumor dissemination *in vivo*. Both agents are able to interrupt the cross-talk between cancer cells and stromal CAFs, although only metformin is able to totally convert the lactate-dependent OXPHOS behavior into a Warburg-like metabolism. From a therapeutic point of view metformin is a very attractive molecule, non-toxic, well tolerated and extremely cheap, and its use should be considered for clinical trials in association with lactate extrusion inhibition (e.g. MCTs inhibitors). Recent clinical trials suggest that metformin could be effective in prostate cancer patients [42, 43], and it is possible that the ability of this drug to interfere with the metabolic circuitry between cancer and stromal cells is the basis of its efficiency. In summary, our study suggests that targeting the PKM2-induced motogen pathways and metabolic coupling in PCa could be a potential therapeutic strategy.

## MATERIALS AND METHODS

### Materials

Unless specified, all reagents were obtained from Sigma and all the antibodies were from Santa Cruz Biotechnology, except for anti-HIF-1α (Becton Dickinson), anti-PKM2 and anti-Src-Tyr416 (Cell Signaling Technology) and anti phospho-Tyr (clone 4G10) (Millipore). HRP-conjugated streptavidin was from Pierce. HIF-1-siRNA and DEC1-siRNA were from Santa Cruz Biotechnology. N-(biotinoyl)-N-(iodoacetyl)ethylenediamine (BIAM) and 2′-7′-dichlorofluoresceindiacetate (H_2_-DCF-DA) were from Molecular Probe. [U-^14^C] lactate and [U-^14^C] glucose were from Perkin Elmer. All the kits used to perform miRNA extraction and quantitative reverse transcriptase PCR were from Qiagen. Lipofectamine 2000 was from Invitrogen. Metformin was obtained by Sigma. The chemical synthesis of the PKM2 activator DASA-58 was kindly performed by Dr. Richichi of the Department of Chemistry (University of Florence). The mitochondrial antioxidant MitoTEMPO was from Santa Cruz Biotechnology. The MCT1 inhibitor, AR-C155858, was from Tocris Bioscience.

### Experimental models

Human PCa cells (PC3, DU145) were obtained and authenticated by PCR/short tandem repeat (STR) analysis from the European Collection of Cell Cultures and maintained at 37°C/5% CO_2_ in DMEM supplemented with 10% fetal bovine serum. Human prostate fibroblasts (HPFs and CAFs) were isolated from surgical explants after patient informed consent, in accord with the Ethics Committee of the Azienda Ospedaliera Universitaria Careggi, as described in [12].

### Fibroblasts and PCa cells activation

HPFs and CAFs were grown to subconfluence and treated for 48 hours with serum-free medium to obtain HPFs CM and CAFs CM, respectively. Alternatively, HPFs were treated for 24 hours with CM from PC3 (obtained culturing PCa cells in serum-free medium for 48 hours) to obtain PCa-activated fibroblasts (PCaAF). PCa cells were treated with the CM collected from fibroblasts (HPFs, CAFs or PCaAFs) for 24 or 48 h.

### Cell transfection

Transient transfections with pSG5c-Src wt, C245A and C487A were performed with Lipofectamine (Invitrogen), following manufacturer's instructions. The efficiency of transfection is 70% and has been evaluated by co-transfection with pEGFP-N1 (ratio pEGFP-N1/pSG5c-Src). Silencing with siRNA was performed with Lipofectamine 2000 following manufacturer's instructions.

### Immunoprecipitation, immunoblot analysis and BIAM labeling

PCa cells (1 × 10^6^) were lysed with RIPA buffer and 500 μg of total proteins were immunoprecipitated. Immunoblot was performed as reported in [8]. For detection of protein redox state, BIAM labeling was performed as previously described [24].

### Preparation of nuclear extract

PCa cells were lysed in (10 mM HEPES pH 7.5, 10 mM KCl, 0.1 mM EDTA, 1 mM dithiothreitol, 0.5% Nonidet-40) for 15 min. After centrifugation (12.000g for 10 min) the supernatant was collected as cytoplasmic extract. The pelleted nuclei were resuspended for 30 min in ice in nuclear extraction buffer (20 mM HEPES pH 7.5,400 mM NaCl, 1 mM EDTA, 1 mM dithiothreitol). Nuclear extract was collected by centrifugation at 12.000 g for 15 min.

### Pyruvate kinase activity

PK activity was measured by a double reactions kinetic assay by lysing cells in 50 mM Tris-HCl pH 7.4, additioned with protease inhibitors and transferred to a TRAP/4 solution (100 mM triethanolamine, 10 mM EDTA, 16 mM MgSO4, pH 7.6) additioned of ADP 100 mM, NADH 10 mM, PEP 40 mM, LDH (Sigma). The absorbance at 340 nm was monitored over 20 min (ε = 6.22 mM^−1^ cm^−1^).

### Assay of intracellular reactive oxygen species (ROS)

To evaluate intracellular production of H_2_O_2_ cells were treated for 3 minute with 5 μg/ml H_2_DCF-DA. After PBS washing, cells were lysed in RIPA buffer and analyzed immediately by fluorimetric analysis at 510 nm. Data have been normalized to total protein content.

### *In vitro* Boyden invasion assay

Invasion assay was performed with 5 × 10^4^ PCa cells on 8-μm-pore Transwells (Corning) coated with 50 μg/cm^2^ of reconstituted Matrigel for 16 h, as described in [9]. Chemotaxis was evaluated by counting the cells migrated to the lower surface of the filters (six randomly chosen fields).

### miRNA analysis

Quantification of miR-205 expression levels was assessed by quantitative reverse transcriptase PCR (qRT-PCR). Total RNA, including small RNAs, was purified using miRNeasy kit. The reverse transcription reaction of 1 μg of total RNA was carried on using miScript II RT kit and the quantification of *miR205* expression level was assessed by Real Time PCR using miScript SYBR Green PCR kit and miScript Primer Assay-HsmiR-205. SNORD61 was used as normalizer (miScript Primer Assay-HsSNORD61, Qiagen). Amplifications were run on 7500 Fast Real-Time PCR System. Data were reported as relative quantity with respect to the calibrator sample using the 2^−ΔΔCt^ method.

### *In silico* binding site analysis

Three human MIR205 [NC_000001 (+) 209602165 – 209605890] files generated by Gene2Promoter analysis (GXP_144577, GXP_144578, GXP_4402679) were used to determine sites of DEC1 binding using Mat Inspector (www.genomatixsuite.de). Putative binding sites for transcription factor were identified using a stringent search setting, with matrix similarity set at 1 and core similarity at 1 (i.e. maximum stringency) to avoid the identification of false positives. The matrix represents the DNA binding profile for DEC1, with the matrix similarity being the quality of a match between the matrix and the input sequence. Core similarity represents the quality of a match between the core sequence of a matrix (the four most conserved positions within a matrix) and the input sequence.

### Glucose and lactate uptake

PCa cells were treated with HPFs CM or CAFs CM for 48 hours, with or without drug administration. Glucose or lactate uptake was evaluated in a buffered solution (140 mmol/L NaCl, 20 mmol/L Hepes/Na, 2.5 mmol/L MgSO4, 1 mmol/L CaCl_2_, and 5 mmol/L KCl, pH7.4) containing 0.5 μCi/mL [U-^14^C] glucose or [U-^14^C] lactate for 15 minutes at 37°C. Cells were subsequently washed with cold PBS and lysed with 0.1 mol/L NaOH. Incorporated radioactive was assayed by liquid scintillation counting and normalized on protein content.

### Detection of released CO_2_ by radioactive lactate

PCa cells were treated with CM for 72 hours and then 0.2 μCi/mL [U-^14^C] lactate was added for 15 minutes. Each dish had a taped piece of Whatman paper facing the inside of the dish wetted with 100 μL of phenyl-ethylamine-methanol (1:1) to trap the CO_2_. Then 200 μL of 4M H_2_SO_4_ was added to cells. Finally, Whatman paper was removed and transferred to scintillation vials for counting.

### Lactate assay

Lactate was measured in the cultured media with Lactate Assay kit (Source Bioscience Life Sciences) according to the manufacturer's instruction.

### Lung colonization assay

Male SCID-bg/bg mice (6–8 weeks old) were injected with PC3 previously conditioned *ex vivo* with CAFs, with or without drug treatment or DEC1 silencing for 48 h. Six mice per group were injected in the lateral tail veins with 1 × 10^6^ PC3 cells, monitored every 3 days and sacrificed after 8 weeks. Lungs were inspected for micrometastases by histological analyses.

### Immunohistochemistry (IHC)

IHC was performed on 29 paraffin-embedded and serially cut prostate cancer or lymph node tissues obtained from St. Joseph's Hospital (Hamilton, Ontario, Canada), following patient consent and approval from the local Ethics Board. Slides were processed and analyzed as previously described [44].

### Statistical analysis

Data are presented as means ± standard deviation from at least three independent experiments. Statistical analysis of the data was performed by unpaired *t*-test. *p*-Values < *p* 0.05 were considered statistically significant.

## SUPPLEMENTARY FIGURES


