# Supplementary material for: Targeting stromal-induced pyruvate kinase M2 nuclear translocation impairs OXPHOS and prostate cancer metastatic spread
Source: Oncotarget. 2015 Jun 27;6(27):24061–74. doi: 10.18632/oncotarget.4448 (PMC4695170; doi:10.18632/oncotarget.4448)
Supplement: Supplementary file 1 [file oncotarget-06-24061-s001.pdf]

SUPPLEMENTARY FIGURES

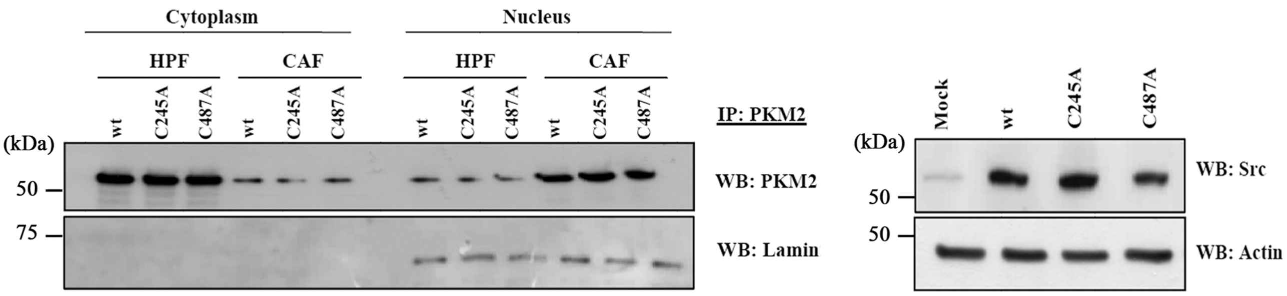

Supplementary Figure S1: Overexpression of Src redox insensitive mutants does not affect nuclear translocation of PKM2. PC3 cells were treated with HPFs CM or CAFs CM for 48 h. The nuclear and the cytosolic compartments were isolated from the whole cell lysates. PKM2 was immunoprecipitated from both the fractions and an anti-PKM2 immunoblot was performed to evaluate PKM2 nuclear translocation. Lamin immunoblot was performed as a control for nuclear extraction **(left panel)**. Src immunoblot was performed as a control of transfection **(right panel)**.

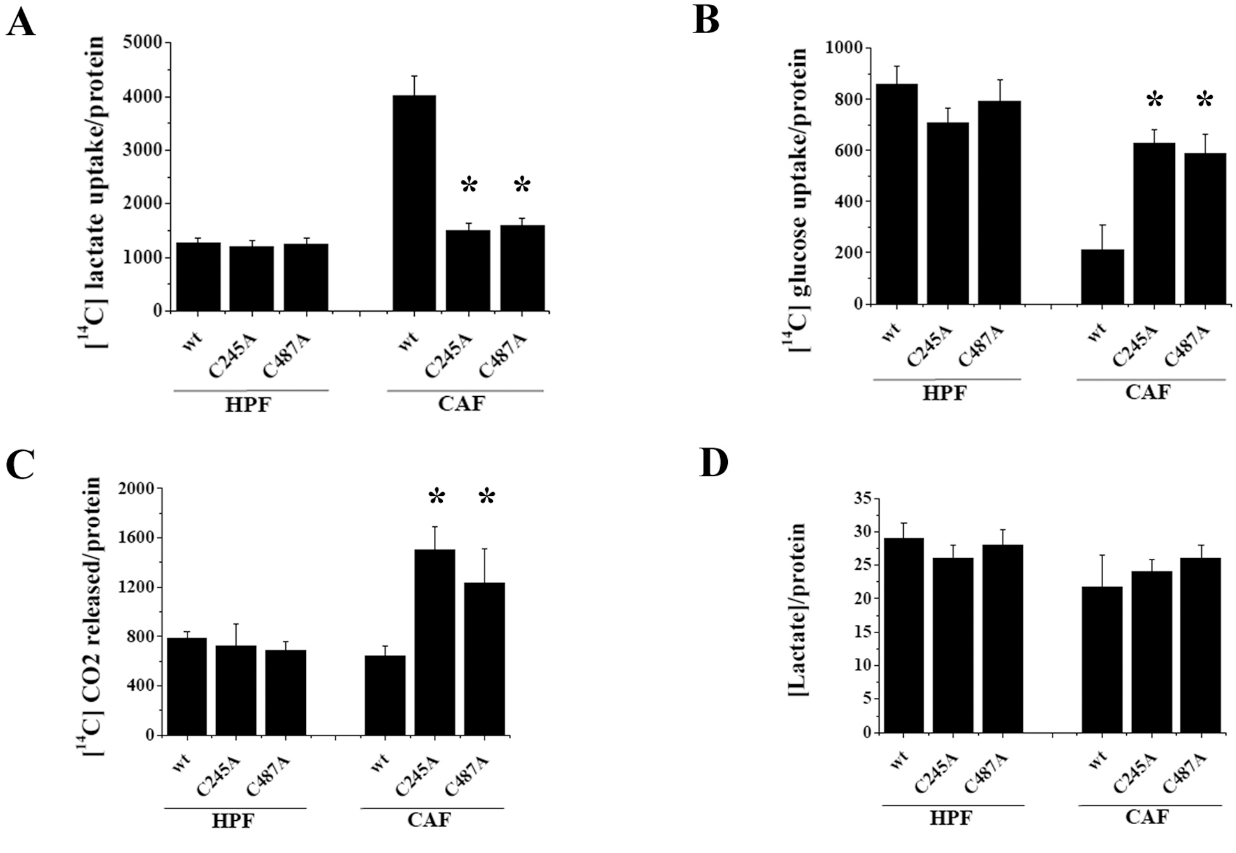

Supplementary Figure S2: Src redox insensitive mutants negatively affect CAF-derived lactate and promote glucose respiration. The redox insensitive Src mutants (C245A and C487A), as well as Src wt, were transfected in PCa cells and after 24 h from transfection, cells were incubated for additional 48 h with HPFs CM or CAFs CM. **A–B.** Evaluation of [^{14}C]-lactate (A) and [^{14}C]-glucose uptake (B) were performed and normalized on protein content. **C**. Respiration of [^{14}C]-glucose was evaluated as [^{14}C]-CO_2 release and normalized on protein content. *$p < 0.005$ *vs* wt CAF **D.** PC3 cells were treated as above, except that after the 48 h of conditioning, cells were refeed with serum-free medium for additional 16 h. The amount of lactate released by PC3 cells into the extracellular medium was quantified and plotted after protein normalization.

A

Matrix	Position from	Position to	strand	Matrix similarity	Sequence*
V$HESF/V$DEC1.02: Basic helix-loop-helix protein known as Dec1, Stra13, Sharp2 or BHLHE40	260	274	(-)	0.809	ccctaaCGTGtggtt
V$HESF/V$DEC1.01: Basic helix-loop-helix protein known as Dec1, Stra13, Sharp2 or BHLHE40	261	275	(+)	0.811	accaCACGttagggg
V$HESF/V$DEC1.01: Basic helix-loop-helix protein known as Dec1, Stra13, Sharp2 or BHLHE40	494	508	(+)	0.800	cctgCAGGtgatcga
V$HESF/V$DEC1.02: Basic helix-loop-helix protein known as Dec1, Stra13, Sharp2 or BHLHE40	876	890	(-)	0.802	tggtctCCTGctccc

***red**: ci-value >60; CAPITALS: core sequence**

B

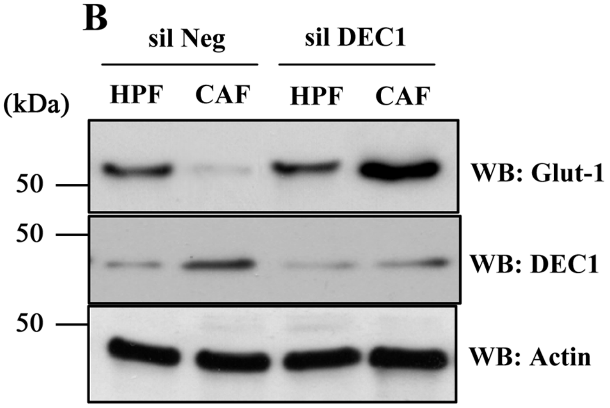

C

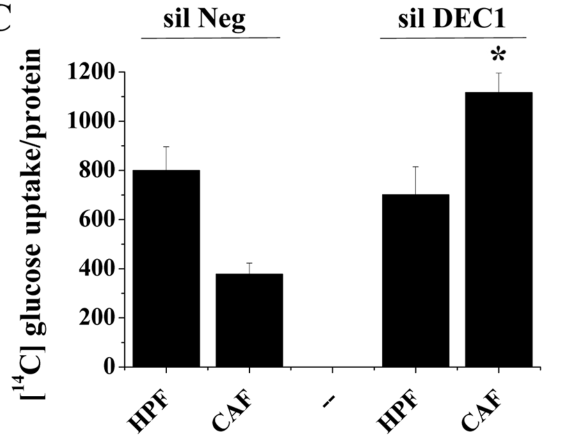

Supplementary Figure S3: The transcriptional repressor DEC1 is involved in CAF-dependent Glut-1 downregulation. **A.** *In silico* binding sites analysis of *GLUT1* promoter region revealed four putative binding sites for DEC1.Two promoter regions identified using Gene2Promoter analysis for human solute carrier family 2 (facilitated glucose transporter), member 1 [GXP_260423 (-) 43396676-43397377 (702 bp) and GXP_3178438 (-) 43424400-43425347 (948 bp)] were used to determine sites of DEC1 binding using Mat Inspector (www.genomatixsuite.de). Putative binding sites for transcription factor were identified using a matrix similarity set at 0.8 and core similarity at 0.75 and applied to Matrix Library 9.2 of Genomatix suite. The matrix represents the DNA binding profile for DEC1, with the matrix similarity being the quality of a match between the matrix and the input sequence. Core similarity represents the quality of a match between the core sequence of a matrix (the four most conserved positions within a matrix) and the input sequence. **B–C.** DEC1 was silenced in PC3 cells and after 24 h cells were incubated for additional 48 h with HPFs CM or CAFs CM (two different siRNA from Santa Cruz Biotechnology and Origene were used, with similar results). The levels of the Glut-1 transporter was evaluated by immunoblotting, using actin as loading control (B). Evaluation of $[^{14}C]$-glucose uptake was performed and normalized on protein content (C). *$p < 0.005$ *vs* control CAF.

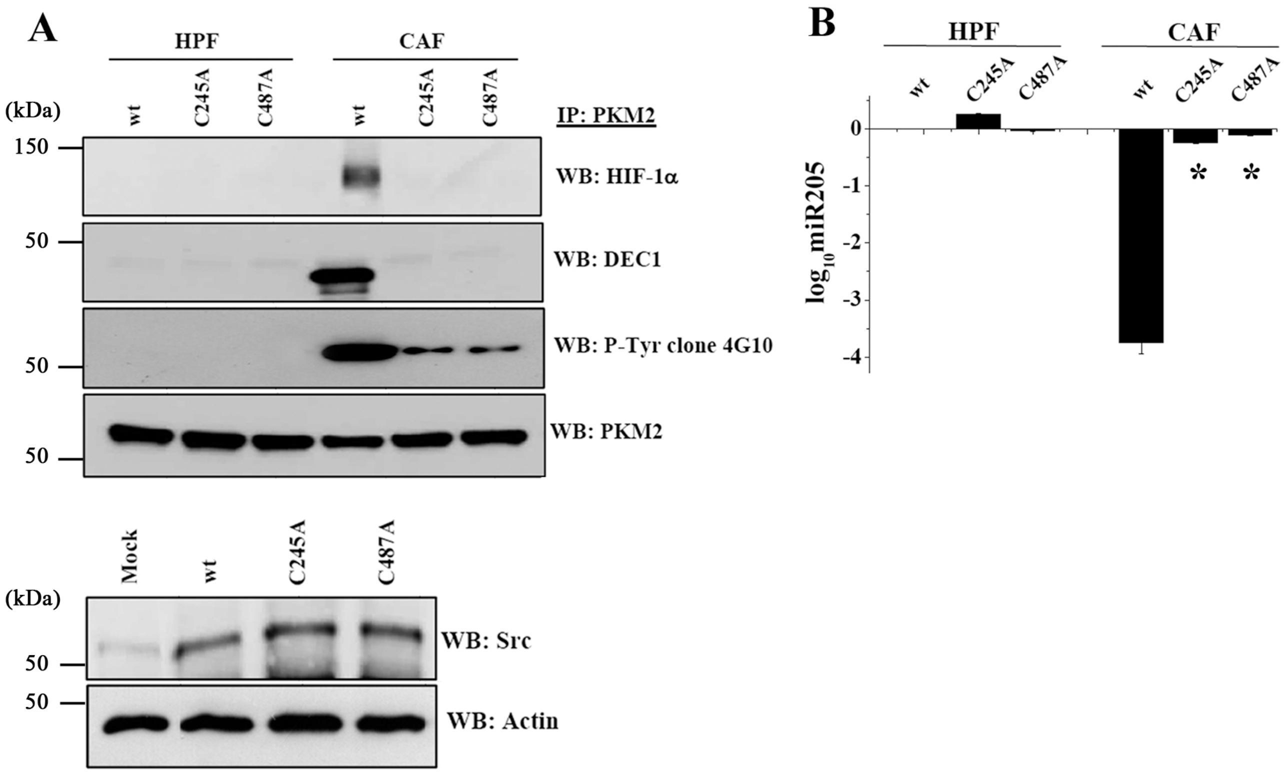

Supplementary Figure S4: Src redox insensitive mutants impair PKM2 association with HIF-1α and DEC-1, hindering miR205 downregulation. **A**. The redox insensitive Src mutants (C245A and C487A), as well as Src wt, were transfected in PC3 cells and after 24 h from transfection, cells were incubated for additional 48 h with HPFs CM or CAFs CM. PKM2 tyrosine phosphorylation and association with HIF-1α and DEC-1 were assessed by means of specific immunoblots on PKM2 immunoprecipitates. PKM2 immunoblot was used for normalization. **B**. qRT-PCR evaluation of miR-205 expression is reported as $\log_{10}$-transformed relative expression with respect to Src wt-transfected PC3 cells treated with HPF-CM.Src immunoblot was performed as a control of transfection. $p < 0.001$ *vs* wt CAF.

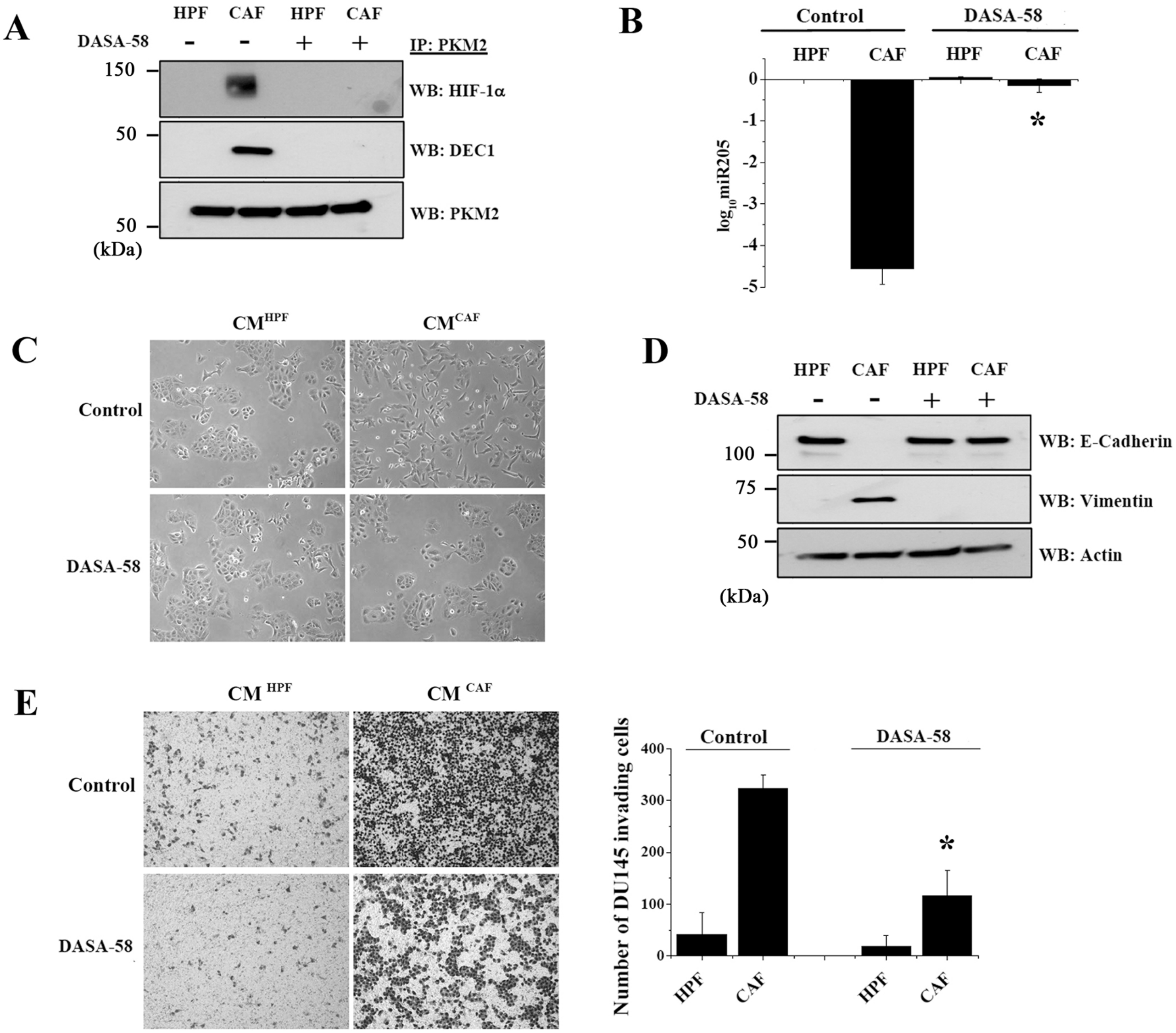

Supplementary Figure S5: Reactivation of PKM2 with DASA-58 impairs its association with HIF-1α and DEC-1, resulting in the inhibition of EMT and invasiveness of DU145 induced by CAFs conditioning. DU145 cells were cultured with CM from HPFs or CAFs for 48 h with or without 40 μM DASA-58. **A**. PKM2 was immunoprecipitated from cell lysates and anti-HIF-1α and anti-DEC-1 immunoblots were performed to evaluate a direct association with PKM2. PKM2 immunoblot was used for normalization. **B.** qRT-PCR analysis of miR-205 expression is shown as $\log_{10}$-transformed relative expression with respect to HPF-CM-DU145 treated cells. *$p < 0.001$ *vs* control CAF **C**. Representative photographs were taken to highlight changes in cell morphology upon CAFs conditioning. **D**. Expression of E-cadherin and vimentin was evaluated by immunoblotting and a WB anti-actin was used as loading control. **E**. An invasion assay on Boyden chamber of DU145 cells treated as indicated was performed. Photographs, representative of six randomly chosen fields, and bar graph for quantification are shown. *$p < 0.005$ *vs* control CAF.

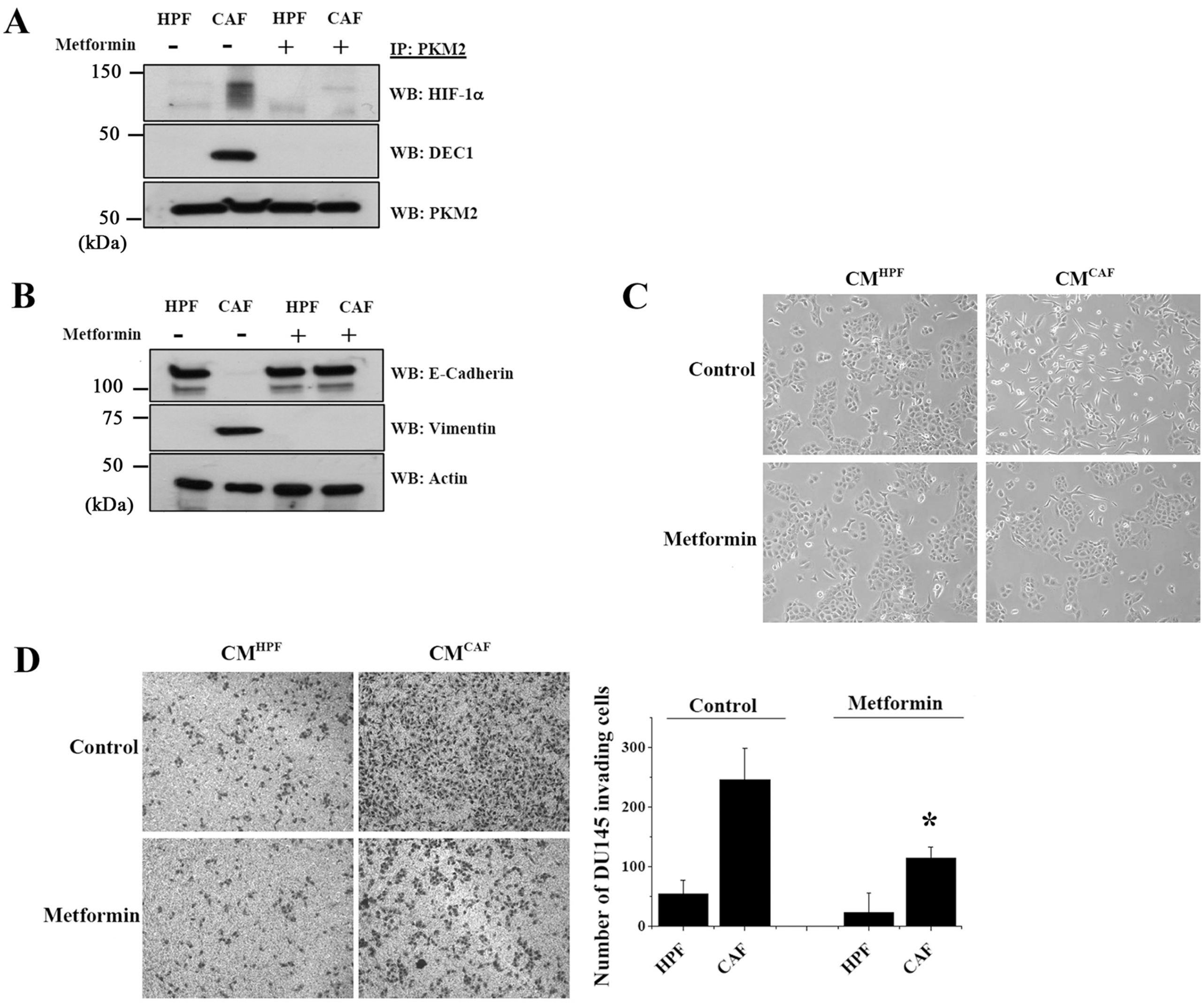

Supplementary Figure S6: Metformin impairs nuclear function of PKM2 and abrogates CAFs dependent EMT and invasiveness of DU145 cells. DU145 cells were cultured with CM from HPFs or CAFs for 48 h with or without 5 mM metformin. **A**. anti-HIF-1α and anti-DEC-1 immunoblots were performed on PKM2 immunoprecipitates to evaluate their direct association with PKM2. PKM2 immunoblot was used for normalization. **B.** Representative photographs were taken to highlight changes in cell morphology upon CAFs conditioning. **C**. Expression of E-cadherin and vimentin was evaluated by immunoblotting and a WB anti-actin was used as loading control. **D**. An invasion assay on Boyden chamber of DU145 cells treated as indicated was performed. Photographs, representative of six randomly chosen fields, and bar graph for quantification are shown. *p < 0.005 *vs* control CAF.
